# Multi-omics integration in autoimmune encephalitis: from novel biomarker discovery to precision therapeutic strategies

**DOI:** 10.3389/fimmu.2026.1893602

**Published:** 2026-07-03

**Authors:** Xudong Chen, Hui Zhang, Shun Ge, Chao Yuan

**Affiliations:** 1 Yancheng First Hospital, Affiliated Hospital of Nanjing University Medical School, Yancheng, China; 2 Hangzhou Adicon Medical Laboratory Center Co., Ltd., Hangzhou, China; 3 Shenzhen Mindray Bio-Medical Electronics Co., Ltd., Shenzhen, China

**Keywords:** autoimmune encephalitis, B-cell immunity, biomarkers, complement activation, immunomics, immunopathogenesis, multi-omics integration, precision therapy

## Abstract

Autoimmune encephalitis (AE) represents a heterogeneous group of disorders characterized by immune-mediated attacks on neuronal antigens within the central nervous system (CNS), leading to diverse clinical manifestations and posing significant challenges in diagnosis and treatment. Recent advances in multi-omics technologies—including genomics, transcriptomics, proteomics, metabolomics, and immunomics—are beginning to provide unprecedented opportunities to unravel the complex immunopathophysiology of AE at a systems level. This review critically examines the application of integrated multi-omics approaches in AE research, with particular emphasis on immunological mechanisms underlying disease pathogenesis. We focus on: (i) the identification of potential diagnostic and prognostic biomarkers with rigorous validation frameworks; (ii) the elucidation of molecular heterogeneity underlying clinical variability, including B-cell clonal expansion, small-cohort evidence for T follicular helper (Tfh) expansion with inferred T follicular regulatory (Tfr) imbalance, and cytotoxic CD8+ T-cell-mediated neuronal injury in paraneoplastic/intracellular-antigen contexts; (iii) the conceptual development of precision medicine strategies tailored to individual immunophenotypic profiles; and (iv) the critical appraisal of current evidence quality and methodological limitations. By synthesizing current findings, this article aims to establish a comprehensive immunological framework that supports the future advancement of personalized therapeutic interventions. The integration of multi-omics data offers a promising conceptual lens through which to understand the intricate immune-neuronal interactions driving AE, though substantial challenges in data standardization, clinical validation, and implementation remain to be addressed.

## 1 Introduction

Autoimmune encephalitis (AE) represents a critical and complex group of inflammatory disorders of the central nervous system (CNS), characterized by immune-mediated neuronal dysfunction that manifests clinically with a spectrum of neuropsychiatric symptoms including cognitive deficits, seizures, behavioral abnormalities, and movement disorders. The immunopathogenesis of AE involves a sophisticated interplay between adaptive and innate immune mechanisms: autoreactive B cells undergo clonal expansion and affinity maturation within germinal centers, facilitated by T follicular helper (Tfh) cells, to produce pathogenic autoantibodies targeting neuronal surface or synaptic proteins such as the N-methyl-D-aspartate receptor (NMDAR), leucine-rich glioma-inactivated 1 (LGI1), and contactin-associated protein-like 2 (Caspr2) (1, 2). Concurrently, cytotoxic CD8+ T cells may directly mediate neuronal injury through perforin-granzyme exocytosis and Fas ligand (FasL)-Fas interactions, particularly in paraneoplastic AE subtypes associated with intracellular antigens (3). Standardized diagnostic criteria established through international consensus efforts (3) have improved clinical recognition, yet a significant subset of patients remains seronegative, posing substantial diagnostic and therapeutic challenges. These antibody-negative AE cases—defined here as patients fulfilling established diagnostic criteria (3) yet lacking detectable serum or cerebrospinal fluid autoantibodies—often exhibit heterogeneous clinical presentations and variable responses to immunotherapy, underscoring the limitations of relying solely on antibody detection for diagnosis and management (5, 6).

The heterogeneity of AE is further complicated by diverse immunopathogenic mechanisms underlying different subtypes. In anti-NMDAR encephalitis, pathogenic autoantibodies disrupt synaptic transmission by targeting the GluN1 subunit, leading to receptor internalization and hypofunction through a mechanism involving cross-linking and endocytosis (2, 7). The F(ab’)2 fragments of these antibodies are sufficient to induce receptor internalization, while the Fc domains may engage complement cascade activation and antibody-dependent cellular cytotoxicity (ADCC), contributing to inflammatory tissue damage (8, 9). In anti-LGI1 encephalitis, autoantibodies alter presynaptic potassium channel function by disrupting the LGI1-ADAM22/23 complex, resulting in increased neurotransmitter release and neuronal hyperexcitability (10, 11). Notably, IgG4-subclass antibodies predominate in LGI1 and CASPR2 encephalitis, which exhibit limited complement activation due to their inability to efficiently bind C1q, suggesting that Fab-mediated functional disruption rather than Fc-mediated effector mechanisms drives pathology in these subtypes (12, 13). Moreover, recent small-cohort single-cell multi-omics studies have suggested that immune dysregulation in AE may extend beyond humoral immunity, involving complex interactions among B cells, CD4+ T cells, CD8+ cytotoxic T cells, and innate immune pathways such as type I interferon and Toll-like receptor (TLR) signaling, which contribute to disease pathogenesis and progression (14, 15).

While a recent review by Zhao et al. (2025) provided a focused perspective on next-generation diagnostic approaches in AE through multi-omics frameworks (1), the present manuscript extends beyond diagnostics to critically examine immunopathogenic mechanisms, biomarker validation frameworks, precision therapeutic stratification strategies, and the methodological limitations facing clinical translation. We specifically emphasize the immunological underpinnings of AE subtypes—including B-cell clonal expansion, reported Tfh expansion with inferred Tfr imbalance, cytotoxic CD8+ T-cell-mediated neuronal injury in paraneoplastic/intracellular-antigen contexts, and complement activation—and provide a critical appraisal of evidence quality that distinguishes our scope from prior work.

Traditional diagnostic modalities, including antibody assays, neuroimaging, and electroencephalography (EEG), while indispensable, have limitations in sensitivity and specificity, particularly in antibody-negative cases (16, 17). Novel imaging biomarkers such as the cortex/striatum metabolic ratio on [18F]-FDG PET and advanced EEG quantitative indices have shown promise in improving diagnostic accuracy and subtype differentiation (18, 19). Additionally, fluid biomarkers reflecting neuroaxonal damage (e.g., neurofilament light chain, NfL), astrocytic activation (e.g., glial fibrillary acidic protein, GFAP), and inflammatory mediators (e.g., soluble CD27) have emerged as valuable tools for disease monitoring, prognostication, and therapeutic response assessment (20, 21). However, these biomarkers require further validation before integration into routine clinical practice (22).

The advent of multi-omics technologies, encompassing genomics, transcriptomics, proteomics, metabolomics, and single-cell analyses, offers unprecedented opportunities to dissect the complex immune-neural interactions in AE at a systems biology level. Integrative multi-omics studies have identified key immunological predictors, such as cerebrospinal fluid (CSF)/serum IgG quotient and lymphocyte subsets, and nominated novel mechanistic players like nucleophosmin (NPM1) that may serve as therapeutic targets (15). Small-cohort single-cell multi-omics profiling has suggested clonal expansion of B cells and activation of interferon and Toll-like receptor pathways in anti-NMDAR encephalitis, providing hypothesis-generating insights into disease pathogenesis and potential intervention points (14). Transcriptomic and machine learning approaches have further delineated immune-related regulatory networks and neuroimmune axes that may support molecular subtyping and future precision medicine strategies (23). Among currently available approaches, integrations that combine molecular biomarkers with clinical, imaging, and electrophysiological data appear closest to potential practical relevance, whereas single-cell and AI-driven integrations remain largely discovery-stage.

The clinical heterogeneity and overlapping features of AE subtypes necessitate precision medicine approaches that integrate multi-omics data with clinical, imaging, and electrophysiological findings to enable accurate diagnosis, prognostication, and personalized therapy. Current immunotherapies, including corticosteroids, intravenous immunoglobulins (IVIg), plasma exchange, and B cell-depleting agents like rituximab, have variable efficacy, particularly in antibody-negative AE, where treatment decisions are often empirical (5, 24). Emerging biomarkers and multi-omics-derived molecular signatures hold promise for guiding therapeutic choices and monitoring treatment response, thereby improving outcomes (1, 21). Furthermore, understanding the molecular mechanisms of antibody-mediated neuronal dysfunction and immune activation can inform the development of targeted therapies, such as agents modulating Fc receptor functions, complement inhibitors, or specific immune pathways (25, 26).

In summary, autoimmune encephalitis is a multifaceted neuroimmunological disorder characterized by significant clinical and molecular heterogeneity. The limitations of antibody-based diagnosis and conventional biomarkers underscore the urgent need for integrative multi-omics approaches to unravel the complex immune-neural networks involved. However, it is essential to recognize that multi-omics integration in AE remains largely at the proof-of-concept stage. Most studies are limited by small sample sizes (frequently n<30), single-center designs, lack of standardized protocols, and insufficient external validation (27). This review critically examines the latest advances in multi-omics integration in AE research, emphasizing immunological mechanisms, biomarker discovery with rigorous validation frameworks, and the conceptual development of precision therapeutic strategies, while critically appraising the maturity of the existing evidence base and the challenges facing clinical translation.

This workflow illustrates a proposed integrative pipeline for autoimmune encephalitis (AE) research and future clinical translation, rather than current standard clinical practice. To visually distinguish evidence maturity, Stage 1 is labeled as established clinical practice, Stages 2 and 3 as discovery-stage components, and Stage 4 as a conceptual or hypothesis-generating precision-medicine framework. Stage 1 encompasses comprehensive clinical phenotyping, including standardized neuropsychiatric assessment, electroencephalography (EEG), magnetic resonance imaging (MRI), and [18F]-fluorodeoxyglucose positron emission tomography ([18F]-FDG PET). Stage 2 involves multi-layered molecular profiling of accessible biospecimens: peripheral blood, cerebrospinal fluid (CSF), and—only in exceptional diagnostic circumstances with unresolved uncertainty after exhaustive non-invasive workup—brain tissue obtained via biopsy. Stage 3 employs bioinformatics integration strategies, including weighted gene co-expression network analysis (WGCNA), multi-omics factor analysis (MOFA+), and machine learning algorithms, to identify molecular signatures and stratify patients into immunophenotypically distinct subgroups: B-cell/antibody-dominant (characterized by IgG1-mediated complement activation and antibody-dependent cellular cytotoxicity [ADCC], e.g., anti-NMDAR encephalitis), T-cell/cytokine-dominant (characterized by cytotoxic CD8+ T-cell-mediated neuronal injury and elevated IL-6/IL-1 signaling), and complement-driven (characterized by elevated C3a/C5a anaphylatoxins and membrane attack complex [MAC] formation). Stage 4 depicts a conceptual decision-support framework wherein molecular stratification guides personalized therapeutic selection: B-cell depletion (rituximab, obinutuzumab) and complement inhibition (eculizumab) for antibody-dominant profiles; cytokine-targeted therapy (tocilizumab, anakinra) for inflammatory phenotypes; and T-cell-directed immunotherapy for cytotoxic CD8+ T-cell-driven subtypes (e.g., paraneoplastic AE with intracellular antigens). Longitudinal biomarker monitoring (NfL, GFAP, CXCL13) enables dynamic assessment of treatment response and relapse risk. The predictive performance metrics shown represent illustrative conceptual benchmarks of reported model performance from published machine learning studies in AE and related neuroinflammatory disorders; these were derived from small, single-center cohorts using cross-validation approaches and should not be interpreted as empirically derived metrics from a unified multi-omics dataset or as validated clinical predictive tools. Currently, most components of this framework remain investigational and have not been implemented in routine clinical practice. Brain biopsy is rarely performed in AE and is reserved for atypical cases where neoplasm, infection, or alternative pathology cannot be excluded by conventional means. Figure created with BioRender.com (licensed academic version) and manually refined by the authors using Adobe Illustrator. No generative artificial intelligence tools were used in the creation of this figure.

## 2 Multi-omics research framework and data integration strategies in autoimmune encephalitis

### 2.1 Core omics technologies and their application scope in AE research

**Figure 1:**
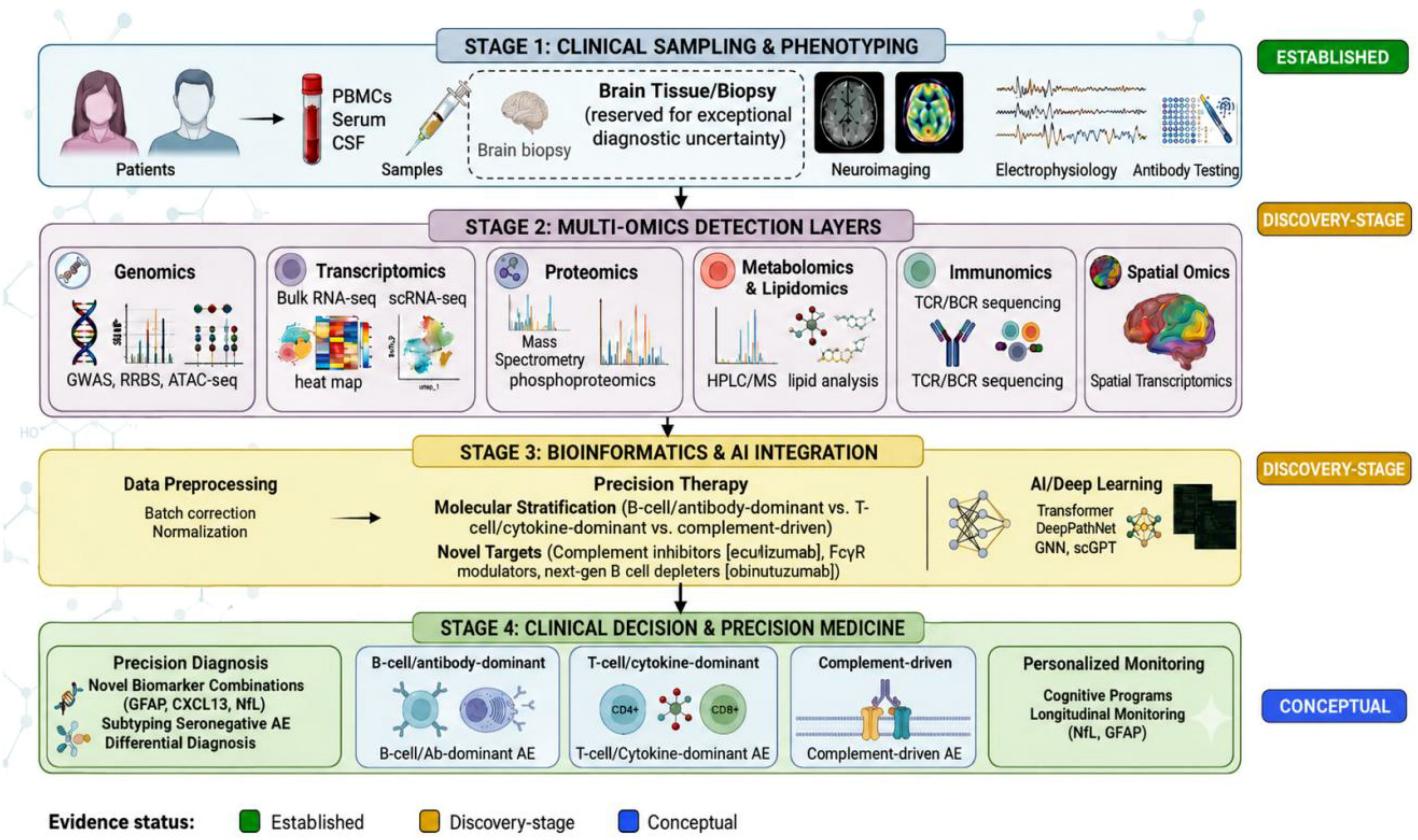
Conceptual precision medicine framework for autoimmune encephalitis based on multi-omics integration.

The investigation of autoimmune encephalitis (AE) through multi-omics technologies has significantly advanced our understanding of its complex immunopathophysiology (**Figure 1**, Stage 1-2), enabling the identification of novel biomarkers and therapeutic targets. Genomics and epigenomics form the foundational layer, where genome-wide association studies (GWAS) have pinpointed susceptibility loci such as the human leukocyte antigen (HLA) region, critical for immune regulation in AE. Whole-genome sequencing further uncovers rare variants that may contribute to disease heterogeneity. Epigenomic analyses, including DNA methylation profiling and chromatin accessibility assays, reveal regulatory modifications influencing gene expression in disease states. For instance, specific methylation patterns have been identified in peripheral immune cells of anti-NMDAR encephalitis patients, suggesting epigenetic dysregulation as a contributor to immune dysfunction, though these findings require replication in independent cohorts (14). In LGI1 antibody encephalitis, Qiao et al. identified aberrant promoter methylation of CSF3, CCL2, and ICAM1 in PBMCs, with methylation levels inversely correlating with gene expression; notably, while PDCD1 expression was reduced and interferon-gamma (IFN-γ) expression elevated, neither exhibited corresponding promoter methylation changes, suggesting that post-transcriptional or alternative epigenetic mechanisms may operate alongside DNA methylation (27). Notably, these methylation-driven genes were directly correlated with clinical grading and prognostic features in the discovery cohort, underscoring their translational relevance for patient stratification.

Transcriptomics, particularly RNA sequencing of cerebrospinal fluid (CSF) immune cells, peripheral blood mononuclear cells (PBMCs), and—only in exceptional diagnostic circumstances—postmortem brain tissue, elucidates differential gene expression and activation of immune pathways such as type I interferon signaling and T cell activation. These transcriptomic signatures may help distinguish AE subtypes and correlate with clinical phenotypes (23). It is important to emphasize that brain biopsy is rarely performed in AE and is generally reserved for exceptional cases with unresolved diagnostic uncertainty where neoplasm, infection, or alternative pathology cannot be excluded by conventional non-invasive testing. Brain tissue multi-omics, while providing the most direct pathophysiological insights, is therefore not a standard component of clinical AE research.

Proteomics, leveraging mass spectrometry, enables unbiased profiling of CSF and serum proteins, facilitating the discovery of novel autoantibody targets and disease-associated proteins including cytokines and synaptic proteins. Phosphoproteomics further delineates aberrant signaling cascades by mapping phosphorylation events, shedding light on intracellular pathway dysregulation (1). Metabolomics and lipidomics analyze small molecules and lipid species in CSF and blood, reflecting metabolic reprogramming, neurotransmitter precursor alterations, and inflammatory states that underpin neuronal dysfunction in AE (28). Immunomics, employing high-throughput T cell receptor (TCR) sequencing, B cell receptor (BCR) sequencing, multiplex cytokine assays, and flow cytometry, provides a granular view of immune cell clonality, subset dynamics, and activation status, directly linking immune phenotypes to clinical manifestations (15). In discovery-stage studies, the integration of scRNA-seq with BCR-seq (scBCR-seq) allows the correlation of BCR clonotypes with cellular states, revealing the relationship between specific clonal expansion and inflammatory phenotypes (29). Collectively, these omics layers offer complementary insights, from genetic predisposition and epigenetic regulation to immune activation and metabolic consequences, forming a comprehensive framework for AE research.

### 2.2 Bioinformatics methods and challenges in multi-omics data integration

Integrating multi-omics datasets in AE research presents both opportunities and challenges (**Figure 1**, Stage 3), necessitating sophisticated bioinformatics strategies to unravel the complex molecular networks underlying disease. Correlation-based integration methods, such as Weighted Gene Co-expression Network Analysis (WGCNA), construct co-expression modules across omics layers, identifying molecular clusters consistently dysregulated in AE (23). WGCNA is a well-established method originally developed for transcriptomic data integration (28). Model-based approaches, including multi-omics factor analysis, decompose heterogeneous data into latent factors representing shared biological signals, facilitating the discovery of cross-omics regulatory modules. MOFA+ represents a statistical framework for comprehensive integration of multi-modal single-cell data (30). Pathway-centric integration maps omics data onto known signaling and gene regulatory pathways, enabling the identification of convergent dysregulated networks implicated in AE pathogenesis (14).

However, challenges such as limited sample sizes, batch effects, and data heterogeneity complicate integration efforts. Small cohorts, common in rare diseases like AE, reduce statistical power and increase overfitting risk. Batch effects arising from different experimental platforms or centers introduce technical variability that can obscure biological signals. To address these, researchers employ robust normalization techniques and leverage public databases to augment datasets, enhancing analytical robustness (1). Machine learning models tailored for small sample sizes, such as random forests and support vector machines, are utilized for feature selection and classification, improving biomarker discovery and patient stratification (15). Systems biology approaches further integrate multi-omics data with protein-protein interaction networks and gene regulatory maps, pinpointing hub molecules that serve as potential therapeutic targets or biomarkers (23). Despite these advances, the complexity of AE immunobiology demands continuous refinement of integrative methodologies, emphasizing the need for standardized protocols, larger multi-center cohorts, and longitudinal data to fully harness multi-omics potential in precision diagnosis and treatment of AE.

### 2.3 Literature search strategy and review scope

This is a narrative review synthesizing published literature on multi-omics integration in autoimmune encephalitis. A comprehensive literature search was conducted using PubMed/MEDLINE, Web of Science, and Embase databases for articles published from January 2010 to May 2026 (date of last search). Search terms included combinations of “autoimmune encephalitis,” “multi-omics,” “genomics,” “transcriptomics,” “proteomics,” “metabolomics,” “immunomics,” “biomarker,” “precision medicine,” and “machine learning.” Inclusion criteria encompassed original research articles, systematic reviews, and meta-analyses examining multi-omics approaches in AE. Case reports with fewer than three patients, non-human studies without direct clinical relevance, and non-English publications without available translations were excluded. Given the rarity of AE and the nascent stage of multi-omics research in this field, formal quality assessment using tools such as GRADE or ROBIS was not performed; instead, we applied a critical appraisal framework emphasizing sample size, study design, replication status, and clinical translation potential (see Section 5). This narrative approach allows for the integration of mechanistic insights across diverse study designs while acknowledging the limitations inherent in the current evidence base.

For interpretive clarity, the evidence discussed throughout this review is considered across three broad maturity levels: (i) clinically anchored evidence, referring to findings supported by reproducible observations across multiple cohorts and already linked to current clinical practice; (ii) reproducible but not yet clinically validated signals, referring to biomarkers or integrated models with biologically coherent support but lacking prospective multi-center validation; and (iii) discovery-stage or hypothesis-generating findings, which include most small-cohort single-cell, multi-layer omics, and AI-driven analyses in AE. This framework is applied below when discussing disease classification, prognosis, mechanistic inference, and treatment stratification.

## 3 Multi-omics-driven biomarker discovery in autoimmune encephalitis

### 3.1 Diagnostic and differential diagnostic biomarkers

**Central versus peripheral biomarker origins.** Biomarkers in AE can be conceptually stratified by their anatomical origin relative to the blood-brain barrier (BBB). CSF-derived markers—including CXCL13, intrathecal IgG synthesis rate, and CSF NfL—more directly reflect CNS-compartmentalized inflammation, B-cell chemoattraction, or neuroaxonal injury, and their elevation may indicate intrathecal production, BBB dysfunction, or both. Peripheral blood markers—such as circulating Tfh cell subsets, serum NfL, and the neutrophil-to-lymphocyte ratio (NLR)—offer more accessible surrogates for longitudinal monitoring but may be confounded by systemic inflammation, infection, age, or comorbid disease. The clinical rationale for this distinction is twofold: CSF markers generally provide higher pathophysiological specificity for CNS-directed autoimmunity, whereas blood markers are more feasible for repeated assessment; meanwhile, the CSF/serum IgG quotient and albumin ratio can serve as operational proxies for BBB integrity and help interpret whether peripheral immune signals are likely to reflect CNS pathology. Future multi-omics integration should therefore explicitly account for central-peripheral compartmentalization, because peripheral and CSF immune profiles are not always concordant in AE.

The identification of diagnostic and differential diagnostic biomarkers in autoimmune encephalitis (AE) is critical, especially for antibody-negative patients who fulfill established diagnostic criteria (3) yet lack detectable serum or cerebrospinal fluid (CSF) autoantibodies. Multi-omics integration, combining proteomic and transcriptomic data from CSF and peripheral blood, has emerged as a proof-of-concept approach to uncover novel biomarker signatures that may aid diagnosis and differential diagnosis. However, it is crucial to emphasize that none of the currently proposed biomarkers are pathognomonic for AE, and their utility lies in combination with clinical assessment rather than as standalone diagnostic tools.

For antibody-negative AE patients fulfilling diagnostic criteria, proteomic analyses of CSF have identified candidate protein combinations such as elevated glial fibrillary acidic protein (GFAP) and chemokine CXCL13, which reflect astrocytic activation and B-cell chemoattraction, respectively. These findings remain investigational and require validation in multi-center cohorts. GFAP is a well-established marker of astrocytic activation across diverse neuroinflammatory and neurodegenerative conditions, including multiple sclerosis, traumatic brain injury, and Alzheimer’s disease; its elevation in AE indicates CNS inflammation but does not establish a specific diagnosis of AE. Similarly, neurofilament light chain (NfL), often discussed in conjunction with GFAP, reflects neuroaxonal injury and is elevated in numerous CNS disorders; in AE, NfL levels correlate with disease severity and may assist in monitoring treatment response, but they are not specific to AE pathophysiology (20, 31). These markers help distinguish AE from other encephalitic conditions only when interpreted within the full clinical context, including neuroimaging, EEG, and antibody testing (1).

Transcriptomic profiling further complements proteomic data by identifying gene expression patterns associated with immune activation and neuroinflammation. Longitudinal multi-omics studies have also identified dynamic biomarker changes correlating with disease activity and subtype stratification. Cytokine panels including interleukin-6 (IL-6), IL-17, and interferon-gamma (IFN-γ) show fluctuating concentrations that parallel clinical severity measured by modified Rankin Scale (mRS) scores and relapse risk. However, IL-6 is a pleiotropic cytokine elevated in systemic inflammation, infection, and malignancy; its presence in CSF indicates inflammatory activity but is not specific to AE. These markers offer a means to monitor disease progression and therapeutic response when integrated with clinical assessment (15). Peripheral blood immune profiling reveals alterations in T cell subsets, such as increased follicular helper T (Tfh) cells, which may serve as accessible surrogates for central nervous system (CNS) immune status. Some studies suggest that certain CSF inflammatory markers may have peripheral blood counterparts with potential diagnostic utility, although further validation is required before blood-based assays can be adopted clinically (14).

Electroencephalography (EEG) and neuroimaging biomarkers also contribute to differential diagnosis. EEG features such as superimposed fast activity and fluctuating abnormalities are more prevalent in anti-NMDAR encephalitis compared to other AE subtypes, aiding subtype differentiation (32). Magnetic resonance imaging (MRI) abnormalities, although less frequent than EEG changes, exhibit subtype-specific patterns; for instance, anti-LGI1 encephalitis often involves the hippocampus and basal ganglia, whereas anti-NMDAR encephalitis shows diffuse cortical hyperintensities (16). The integration of multi-omics data with clinical, electrophysiological, and imaging findings enhances diagnostic accuracy, particularly in antibody-negative cases where conventional antibody testing is inconclusive. Nevertheless, the technological complexity, cost, and need for validation in larger cohorts represent significant barriers to clinical translation (33). These subtype-specific biomarker profiles are summarized in **Table 1**.

**Table 1:** Multi-omics biomarker profiles and effector mechanisms across AE subtypes.

Biomarker category	Anti-NMDAR encephalitis	Anti-LGI1 encephalitis	Antibody-negative AE
Genomic/Epigenomic	HLA region association (1)	Promoter methylation of CSF3, CCL2, ICAM1 (inverse correlation with PBMC expression); serum PDCD1↓/ICAM1↑ prognostic signature (27)	Not established in current literature
Transcriptomic	Type I interferon signature, B cell clonal expansion, Tfh cell activation (14)	Kv1 channel-related gene expression (10)	Innate immune activation, elevated NLR and neutrophilia (34)
Proteomic	CXCL13↑, GFAP↑, NfL↑, IgG1 predominant (FcγR-mediated ADCC/CDC) (1, 8, 20, 25, 26)	LGI1 antibody-mediated synaptic protein alterations, IgG4 predominant (Fab-mediated functional disruption, limited complement activation) (10, 12)	YKL-40↑, S100B↑, IL-6↑ (22)
Metabolomic	Not established in current literature	Not established in current literature	Not established in current literature
Immunomic	Plasma cell expansion, Tfh cell↑, inferred Tfr imbalance (14, 15, 44)	Not established in current literature	NK cell dysfunction, activated monocyte signatures (15)
Effector Mechanism	FcγR-mediated ADCC/CDC, complement activation (C1q-C3-C5), NMDAR internalization (2, 7–9)	Fab-mediated Kv1 channel disruption, limited complement activation (IgG4 structural constraints) (10–13)	Heterogeneous; no single established effector mechanism; innate immune activation signatures reported, with T cell cytotoxicity considered mainly when paraneoplastic or intracellular-antigen AE is suspected (3, 5, 40)

a. Biomarkers listed are not pathognomonic for AE and may be elevated in other neuroinflammatory, neurodegenerative, or infectious conditions. Their utility lies in longitudinal monitoring and severity assessment within the full clinical context.

b. Metabolomic and epigenomic profiles in AE remain underinvestigated and require further validation in independent cohorts.

c. GFAP and NfL reflect astrocytic activation and neuroaxonal injury, respectively, across diverse CNS pathologies; IL-6 is a pleiotropic cytokine elevated in systemic inflammation and infection.

d. Epigenetic findings in anti-LGI1 encephalitis are derived from a single-center study (n=20) using PBMCs; CNS tissue epigenetic profiles remain uncharacterized.

e. Effector mechanism distinctions are based on emerging immunological evidence and require prospective validation in multi-center cohorts. IgG1 and IgG4 subclass distributions represent predominant patterns rather than absolute exclusivity.

f. Tfr imbalance in anti-NMDAR encephalitis is inferred from Tfh expansion patterns and germinal center dysregulation; experimentally validated Tfr dysfunction in specific AE subtypes remains to be established.

g. Most multi-omics data in AE are derived from anti-NMDAR cohorts; profiles for anti-LGI1, CASPR2, GAD65-associated, and paraneoplastic AE remain fragmentary and should not be assumed to be interchangeable.

### 3.2 Prognostic and treatment response predictive biomarkers

Prognostic biomarkers and predictors of treatment response are essential for optimizing therapeutic strategies in autoimmune encephalitis (AE), particularly given the variable clinical course and heterogeneous treatment outcomes. Multi-omics integration has enabled the exploratory development of predictive models that combine baseline clinical features, antibody profiles, genomic risk loci, and immune cell phenotypes to forecast patient responses to first-line immunotherapies such as corticosteroids, intravenous immunoglobulin (IVIg), and plasma exchange. Machine learning algorithms applied to these integrated datasets have identified key immunological indicators including cerebrospinal fluid (CSF)/serum IgG quotient, lymphocyte subsets (double negative and double positive T cells), natural killer (NK) cell counts, and T cell percentages that predict treatment responsiveness with reported area under the curve (AUC) values up to 0.978 (15). However, it is critical to contextualize these metrics: this reported AUC was derived from a single-center cohort of 91 patients using leave-one-out cross-validation. Independent external validation in multi-center cohorts with larger sample sizes and diverse ethnic populations is essential before clinical translation can be considered. If externally validated, such models could facilitate early identification of patients likely to be refractory to first-line therapies who may benefit from second-line agents like rituximab or cyclophosphamide, thereby supporting more individualized treatment escalation.

Long-term neurological outcomes, including cognitive function and psychiatric sequelae, have been explored using composite biomarker panels that integrate acute-phase CSF metabolomics and recovery-phase neuroimaging features. Metabolomic profiling may identify neurodegeneration-associated metabolites whose levels correlate with neuronal injury, while advanced imaging modalities can capture structural and functional brain changes associated with cognitive decline and psychiatric symptom persistence. These multimodal biomarkers provide a potential framework for monitoring disease evolution and tailoring rehabilitation strategies, though their prognostic accuracy requires prospective validation (20). In paraneoplastic AE, multi-omics analyses integrating tumor transcriptomics with immune profiling have suggested biomarkers potentially associated with tumor risk, such as gene expression signatures in ovarian teratomas and thymomas linked to autoimmune responses (23). Such observations may inform future tumor-screening and surveillance strategies, but they require validation before being used to guide management. Additionally, soluble biomarkers such as intrathecal IgG synthesis rate and CSF albumin concentration have been evaluated as predictors of poor immunotherapy response, with combined biomarker models outperforming individual markers in prognostic accuracy (reported AUC up to 0.896) (30). This metric should be interpreted cautiously given its derivation from a retrospective analysis of 53 patients with heterogeneous AE subtypes and the inherent risk of overfitting in small cohorts. Peripheral blood biomarkers, including elevated neutrophil-to-lymphocyte ratio (NLR) and neutrophil counts, have emerged as candidate predictors of seizure risk in AE (34).

Representative forms of multi-omics integration and their current practical relevance in AE are summarized in **Table 2**.

**Table 2:** Representative multi-omics integration approaches in AE and their current practical relevance.

Integration modality	Main application	Representative example/AE context	Current evidence maturity and critical gap
CSF/serum proteomics + clinical/EEG/MRI data	Adjunctive diagnosis/differential diagnosis	Candidate inflammatory and neuronal injury biomarkers (e.g., GFAP, NfL, CXCL13) interpreted with imaging and EEG, especially in antibody-negative AE	Most clinically informative at present, but still lacks prospective multi-center validation; requires harmonized assays and explicit interpretation of BBB integrity.
Clinical features + immune phenotyping + machine learning	Treatment-response prognosis	Prediction of first-line immunotherapy response using CSF/serum IgG quotient, T-cell subsets, and NK-cell measures	Exploratory; derived mainly from small, single-center cohorts; lacks prospective validation in antibody-negative or subtype-specific cohorts.
scRNA-seq + BCR/TCR sequencing	Molecular subtype and mechanism discovery	B-cell clonal expansion, Tfh enrichment, and CD8+ T-cell programs in anti-NMDAR or paraneoplastic AE	Discovery-stage and hypothesis-generating; requires multi-center replication because clonal expansions may be patient-specific and not generalizable.
Transcriptomics + pathway/network analysis (e.g., WGCNA, MOFA+, ML)	Biological stratification/candidate target discovery	Interferon/TLR pathway activation and immune-regulatory networks in anti-NMDAR encephalitis	Discovery-stage; biologically informative, but not yet clinically actionable; requires functional validation and testing beyond anti-NMDAR cohorts.
Tumor transcriptomics + immune profiling	Paraneoplastic risk stratification	Ovarian teratoma- or thymoma-associated AE	Limited and subtype-specific; currently exploratory; tumor-linked signatures require prospective validation.

Despite these advances, challenges persist in standardizing biomarker assays, validating predictive models across diverse populations, and integrating multi-omics data into routine clinical workflows. Future research should focus on longitudinal multi-omics studies to capture dynamic biomarker changes, refine predictive algorithms, and explore novel therapeutic targets such as nucleophosmin (NPM1), which has been implicated in AE pathogenesis through integrated single-cell transcriptomics and spatial transcriptomics (15). Overall, multi-omics-driven biomarker discovery holds promise for precision medicine in AE by enabling early prognostication, individualized treatment selection, and improved long-term outcomes.

## 4 Pathophysiological insights from multi-omics integration

### 4.1 Molecular network analysis of immune-neural interactions

The pathogenesis of autoimmune encephalitis (AE) is intricately linked to immune responses targeting synaptic proteins, with multi-omics integration shedding light on the molecular networks underlying immune-neural interactions. Central to this process is the initiation and amplification of autoimmune responses against synaptic proteins such as N-methyl-D-aspartate receptor (NMDAR) and leucine-rich glioma-inactivated 1 (LGI1). The generation of pathogenic autoantibodies involves a sophisticated germinal center (GC) reaction wherein autoreactive B cells undergo somatic hypermutation (SHM) and affinity maturation under the direction of T follicular helper (Tfh) cells (35, 36). Tfh cells, characterized by expression of Bcl-6, CXCR5, and PD-1, provide critical co-stimulatory signals to GC B cells through CD40L-CD40 interactions and IL-21 secretion, promoting B cell proliferation, class-switch recombination, and differentiation into antibody-secreting plasma cells (35). The balance between Tfh cells and T follicular regulatory (Tfr) cells—Foxp3+ Tregs that express Tfh-associated markers and migrate into GCs to suppress excessive B cell responses—is critical for maintaining immunological tolerance. Expansion of autoreactive Tfh clones has been reported in AE through small single-cell transcriptomic and immunophenotyping studies (14, 15). Tfr cell imbalance has been inferred from observed Tfh expansion and germinal center dysregulation in small-cohort studies (36, 37), but direct experimental validation of Tfr cell dysfunction in specific AE subtypes remains a discovery-stage goal for future studies. (**Figure 1**, Stage 2-3).

Multi-omics data suggest that molecular mimicry, exemplified by epitope similarity between viral antigens like herpes simplex virus and synaptic proteins, may trigger initial autoimmunity. This is supported by experimental evidence demonstrating that asymptomatic HSV-1 infection can exacerbate experimental autoimmune encephalomyelitis, a model of neuroinflammation, indicating a viral contribution to disease onset and severity (38). Epitope spreading further expands the autoimmune repertoire, while the local inflammatory milieu within the central nervous system (CNS) is shaped by activated microglia and astrocytes. These glial cells secrete cytokines and chemokines that modulate immune cell recruitment and activation, creating a feedback loop that sustains inflammation. Small-cohort single-cell multi-omics analyses have suggested clonal expansion of B cells, particularly plasma cells, in anti-NMDAR encephalitis, with type I interferon (IFN-I) pathway activation enhancing antibody production (14). Toll-like receptor 2 (TLR2) activation in myeloid cells further contributes to tumor necrosis factor-alpha (TNF-α) secretion, promoting adaptive immune responses. B-T cell interactions have been investigated through single-cell transcriptomics combined with T-cell receptor (TCR) and B-cell receptor (BCR) sequencing, suggesting that antigen-specific T follicular helper cells may facilitate B cell clonal expansion, affinity maturation, and differentiation into antibody-secreting plasma cells within germinal centers (15). This coordinated cellular interplay remains mechanistically plausible but requires larger, subtype-specific validation.

Concurrently, blood-brain barrier (BBB) dysfunction is a hallmark of AE pathophysiology. Integrated proteomic and transcriptomic analyses identify aberrant expression of endothelial tight junction proteins and adhesion molecules, such as ICAM-1 and VCAM-1, which facilitate peripheral immune cell transmigration into the CNS (1). Chemokine gradients established by CNS-resident cells further guide immune cell infiltration. These molecular insights collectively delineate a complex network wherein synaptic protein-targeted autoimmunity is initiated by molecular mimicry and epitope spreading, amplified by glial-mediated inflammation, and sustained through B-T cell cooperation and BBB disruption. Understanding these interconnected pathways provides a foundation for developing targeted therapies aimed at interrupting key nodes in the immune-neural interface in AE (**Figure 1**, Stage 2-3).

Epigenetic modifications, including alterations in DNA methylation patterns of immune-related genes, have been proposed as potential contributors to immune dysregulation in autoimmune encephalitis, though specific methylation signatures require independent validation in larger cohorts (14). Notably, interferon-gamma (IFN-γ) signaling pathways have been implicated in AE pathogenesis through transcriptomic analyses, representing an important cytokine-mediated mechanism that warrants further epigenetic investigation.

### 4.2 Cellular and humoral effector mechanisms in AE

The immunopathogenic mechanisms of AE involve distinct effector pathways that vary according to antibody subclass, antigen localization, and disease subtype. Autoantibodies targeting neuronal cell surface antigens (NSAbs), such as NMDAR and LGI1, exert pathogenic effects primarily through their antigen-binding (Fab) domains. In anti-NMDAR encephalitis, patient-derived IgG antibodies induce cross-linking and internalization of NMDARs, reducing synaptic surface expression and impairing glutamatergic neurotransmission (7, 39). This mechanism is reversible upon antibody removal, as demonstrated in passive transfer models where CSF containing NMDAR antibodies induces reversible behavioral and memory deficits in mice (2). The pathogenicity of NSAbs is further modulated by their Fc domains, which can engage activating Fc gamma receptors (FcγRI, FcγRIII) on microglia and natural killer (NK) cells, triggering antibody-dependent cellular cytotoxicity (ADCC) and complement-dependent cytotoxicity (CDC) through classical pathway activation (8, 9). However, the predominant IgG4 subclass in LGI1 and CASPR2 encephalitis exhibits limited complement activation due to structural constraints in C1q binding, suggesting that Fab-mediated functional disruption rather than Fc-mediated effector mechanisms drives pathology in these subtypes (12, 13).

In contrast to surface-targeting antibodies, autoantibodies directed against intracellular neuronal antigens (e.g., Hu, Yo, Ma2) are generally considered non-pathogenic in isolation, as they cannot access their intracellular targets under physiological conditions. Instead, the pathophysiology of paraneoplastic AE with intracellular antibodies is primarily driven by cytotoxic CD8+ T cells that recognize peptides derived from these antigens presented on major histocompatibility complex class I (MHC I) molecules on neuronal surfaces (3, 4). Upon antigen recognition, CD8+ T cells form immunological synapses with target neurons and induce cell death through two principal pathways: (i) perforin-granzyme exocytosis, wherein perforin creates membrane pores allowing granzyme entry and caspase-mediated apoptosis; and (ii) Fas ligand (FasL)-Fas interaction, triggering death receptor-mediated apoptosis (40, 41). These cytotoxic CD8+ T-cell-mediated mechanisms are predominantly implicated in paraneoplastic AE subtypes associated with intracellular antigens, whereas surface antibody-mediated mechanisms (e.g., NMDAR internalization) dominate in NSAb-associated AE. The choice between cytotoxic pathways depends on the strength of T cell receptor (TCR) signaling: strong antigen signals favor perforin-mediated killing, while weak signals preferentially activate the FasL-Fas pathway (40). Notably, CD8+ T cell attack can also induce collateral damage to neighboring neurons through glutamate excitotoxicity and cytokine-mediated neuroinflammation, even in the absence of direct antigen recognition (40).

In small cohorts, recent single-cell transcriptomic and TCR sequencing studies have suggested oligoclonal expansion of neuron-reactive CD8+ T cells in the blood, CSF, and brain tissue of patients with paraneoplastic AE, suggesting antigen-driven clonal proliferation (9). These CD8+ T cells display an encephalitogenic transcriptional program characterized by elevated expression of cytotoxic effector molecules (perforin, granzyme B), pro-inflammatory cytokines (IFN-γ, TNF-α), and chemokine receptors (CXCR3, CCR5) that facilitate CNS trafficking (9). The coexistence of humoral and cellular immune responses in certain AE subtypes—such as anti-NMDAR encephalitis with ovarian teratomas, where both pathogenic antibodies and cytotoxic T cells may target shared neuronal antigens—highlights the complexity of immunopathogenic mechanisms and the need for multi-omics approaches to dissect their relative contributions (9).

Complement activation represents another critical effector mechanism in AE pathophysiology. Systematic profiling of complement proteins in immunotherapy-naive AE patients has demonstrated elevated CSF levels of activated complement components (C3a, C4a, C5a) compared to healthy controls and multiple sclerosis patients (40). This intrathecal complement activation is observed in both NSAb-associated and GAD65-antibody-associated encephalitis, suggesting that complement-mediated tissue damage contributes to neuronal injury across AE subtypes. The anaphylatoxin C5a, generated during terminal complement activation, functions as a potent chemoattractant for neutrophils and monocytes, while the membrane attack complex (MAC, C5b-9) can directly induce neuronal lysis (40). Intravenous immunoglobulin (IVIg), a first-line therapy for AE, exerts therapeutic effects in part through complement inhibition—specifically, IVIg contains natural antibodies that suppress complement activation and prevent MAC formation, thereby protecting neuronal integrity (42, 43). The efficacy of rituximab, a B cell-depleting anti-CD20 monoclonal antibody, involves complement-dependent cytotoxicity (CDC) as a primary mechanism of B cell elimination, though variable responses may relate to Fcγ receptor polymorphisms, BAFF levels, and the presence of circulating immune complexes that can interfere with antibody-mediated depletion (40)(**Figure 1**, Stage 3).

### 4.3 Molecular basis of clinical heterogeneity in autoimmune encephalitis

Although anti-NMDAR encephalitis currently dominates the multi-omics literature, the applicability of these approaches to other AE subtypes deserves explicit consideration. In anti-LGI1 and CASPR2 encephalitis, available evidence remains relatively sparse and is weighted toward targeted epigenetic, proteomic, or functional studies rather than fully integrated multi-layer datasets. For example, anti-LGI1 and CASPR2 encephalitis are typically associated with IgG4-predominant, Fab-mediated functional effects, whereas anti-NMDAR encephalitis more often involves IgG1-predominant antibody responses with potential Fc-mediated effector mechanisms (10–14). Whether peripheral B-cell clonal expansion, intrathecal plasma-cell signatures, and Tfh/Tfr patterns in IgG4-predominant AE resemble those reported in anti-NMDAR cohorts remains insufficiently compared. In GAD65-associated and paraneoplastic/intracellular-antigen AE, mechanistic relevance may be high—particularly for T-cell and complement-related pathways—but cohort heterogeneity and small sample sizes limit generalizable biomarker discovery. Antibody-negative AE represents another potentially important setting for multi-omics application because conventional serology is uninformative, yet the biological heterogeneity of these cases complicates interpretation. Accordingly, findings derived from anti-NMDAR cohorts should not be assumed to generalize across all AE subtypes without subtype-specific validation.

The clinical heterogeneity observed in autoimmune encephalitis (AE) is underpinned by distinct molecular signatures associated with antibody subtypes, patient age, and comorbid conditions, as revealed by multi-omics analyses. Comparative cerebrospinal fluid (CSF) multi-omics profiling of patients with anti-NMDAR and anti-LGI1 encephalitis has suggested unique immune activation patterns and neurodegeneration markers that correlate with divergent clinical phenotypes. For instance, anti-NMDAR encephalitis is characterized by diffuse cortical and subcortical T2 FLAIR hyperintensities on MRI and EEG features such as extreme delta brush and frontotemporal delta activity, reflecting widespread neuronal dysfunction (16, 32). In contrast, anti-LGI1 encephalitis often presents with focal hippocampal or basal ganglia involvement and faciobrachial dystonic seizures, linked to presynaptic Kv1 channel loss and action potential broadening, as elucidated by electrophysiological and super-resolution microscopy studies (10, 11). These subtype-specific molecular and electrophysiological profiles explain differences in symptomatology, such as psychiatric manifestations in anti-NMDAR versus limbic seizures in anti-LGI1 AE.

Epigenetic differences further contribute to subtype heterogeneity. In LGI1 encephalitis, Qiao et al. identified distinct PBMC methylation patterns at the promoters of CSF3, CCL2, and ICAM1, with methylation levels inversely correlating with PBMC expression (27). Interestingly, interferon-gamma (IFN-γ) exhibited elevated PBMC expression without corresponding promoter methylation changes, and PDCD1 showed reduced expression despite unchanged promoter methylation, suggesting that DNA methylation is not the sole epigenetic mechanism regulating immune gene expression in this subtype (27). The prognostic value of decreased serum PDCD1 combined with increased ICAM1 further underscores the clinical relevance of these epigenetic-immune axes, though these findings are limited by small sample sizes (n=20 for RRBS; n=5 for pyrosequencing validation) and single-center design (27).

Age-related molecular differences further contribute to heterogeneity. Pediatric AE patients, particularly those antibody-negative, exhibit higher neutrophil-to-lymphocyte ratios and more severe neurological impairment compared to antibody-positive counterparts, suggesting developmental immunological distinctions influencing disease susceptibility and progression (44). Cognitive outcomes also differ, with antibody-negative pediatric AE associated with poorer executive function and higher rates of postencephalitic epilepsy relative to NMDAR antibody-positive cases (44). These findings underscore the impact of immune maturation and neurodevelopmental stage on disease expression. Comorbidities such as tumors and infections modulate AE molecular landscapes and clinical trajectories. Paraneoplastic AE subtypes exhibit elevated neurofilament light chain and total tau levels, indicating more pronounced neuroaxonal injury (21). Moreover, coexisting infections like HSV-1 may precipitate or exacerbate AE by molecular mimicry and immune activation (38). The presence of malignancy or infection reshapes immune responses, influencing antibody profiles, cytokine milieu, and BBB integrity, thereby altering disease course and therapeutic responsiveness. Collectively, multi-omics integration reveals that AE clinical heterogeneity arises from antibody subtype-specific immune-neural interactions, age-dependent immunological and neurodevelopmental factors, and the modifying effects of comorbid conditions. These molecular distinctions may inform future precision medicine approaches, although tailored diagnostics and individualized treatment strategies require further prospective validation before routine clinical use.

## 5 Precision therapeutic strategies guided by multi-omics integration

### 5.1 Molecularly stratified personalized therapeutic pathways

To distinguish current clinical practice from future precision-medicine aspirations, it is important to separate established AE therapies from hypothesis-generating multi-omics-guided strategies. Established treatment frameworks currently rely on clinical diagnosis and conventional biomarkers and include first-line corticosteroids, intravenous immunoglobulin (IVIg), plasma exchange, and commonly used second-line agents such as rituximab and cyclophosphamide. By contrast, molecularly stratified therapeutic pathways informed by multi-omics profiling remain conceptual (**Figure 1**, Stage 4).

Within this conceptual framework, patients with cytokine-dominant inflammatory phenotypes marked by elevated pro-inflammatory cytokines such as IL-6 and IL-1 may theoretically be candidates for cytokine-targeted therapies, including tocilizumab (anti-IL-6 receptor) or anakinra (IL-1 receptor antagonist) (1, 45–47). However, although tocilizumab has been evaluated in an institutional cohort and small pediatric case series in refractory AE, and anakinra has been reported mainly in individual refractory encephalitis/status epilepticus cases, no randomized or prospective multi-omics-stratified trial has validated the superiority of these agents over standard immunotherapy in specific AE molecular subtypes (45–47). While these molecular stratification approaches are conceptually compelling, current validation is limited to small, single-center cohorts, and prospective multi-center trials are required before any clinical implementation can be considered.

Longitudinal monitoring of immune cell subsets and cytokine profiles through simplified multi-omics panels may eventually allow dynamic adjustment of treatment intensity, helping optimize immunosuppression to balance efficacy and safety. For example, tracking specific cytokine combinations or immune cell subpopulations could inform the tapering or escalation of immunotherapy, minimizing risks of under-treatment or excessive immunosuppression (15). This approach is particularly valuable given the variable disease activity and relapse risk in AE. Moreover, integrating molecular stratification with clinical and imaging biomarkers could enhance the precision of therapeutic decisions if validated in prospective studies. For example, patients with predominant T cell activation and neuroinflammation may require adjunctive therapies targeting T cell pathways or neuroprotective agents. If validated, molecularly guided personalized treatment pathways could represent a shift from empirical immunotherapy toward precision medicine in AE; at present, however, this remains a research framework rather than an established clinical approach.

### 5.2 Discovery of novel therapeutic targets and drug repurposing

Multi-omics integration has emerged as a conceptual strategy to identify novel therapeutic targets in autoimmune encephalitis by elucidating key molecular drivers within pathogenic networks (**Figure 1**, Stage 4). Systems-level analyses combining transcriptomics, proteomics, and single-cell sequencing have pinpointed critical nodes such as aberrantly activated complement components, microglial activation pathways, and transcription factors that orchestrate neuroinflammation (15). For example, dysregulated complement activation and microglial-mediated neurotoxicity have been implicated in AE pathogenesis, suggesting that complement inhibitors (e.g., eculizumab, a C5 monoclonal antibody approved for paroxysmal nocturnal hemoglobinuria and atypical hemolytic uremic syndrome) or microglial modulators could theoretically serve as innovative therapeutic agents (1, 40). Furthermore, computational drug repositioning approaches leverage disease-specific gene expression signatures and protein interaction networks to nominate existing drugs that may reverse pathogenic molecular profiles. By comparing AE-associated molecular signatures with drug-induced perturbation databases, candidate compounds such as Janus kinase (JAK) inhibitors and sphingosine-1-phosphate (S1P) receptor modulators, currently approved for other autoimmune diseases, have been proposed for AE subtypes characterized by specific immune dysregulation (23). These nominations are algorithmic predictions based on gene expression correlations; they require experimental validation in AE-specific cellular and animal models, followed by structured clinical trials, before any therapeutic relevance can be claimed. Thus, drug-repositioning analyses should be regarded as hypothesis-generating tools that prioritize candidates for preclinical testing rather than as evidence for immediate clinical use.

Additionally, multi-omics data may facilitate the identification of novel biomarkers predictive of therapeutic response, potentially enabling future stratification of patients who might benefit from repurposed agents. For instance, patients exhibiting upregulation of JAK-STAT signaling pathways could be considered candidate subgroups for future evaluation of JAK inhibitors, while those with aberrant S1P receptor signaling could be evaluated for modulators like fingolimod (23). The development of next-generation B cell-targeting therapies offers another promising avenue. Obinutuzumab, a glycoengineered type II anti-CD20 monoclonal antibody with enhanced antibody-dependent cellular cytotoxicity (ADCC) and reduced internalization compared to rituximab, has demonstrated superior B cell depletion in preclinical models and may overcome resistance mechanisms observed with rituximab in autoimmune diseases (40). Similarly, targeting B cell activating factor (BAFF) with belimumab or targeting CD19 with inebilizumab represent potential alternative strategies for refractory cases, but AE-specific validation remains necessary (40). Overall, the integration of multi-omics-driven target discovery with computational drug repositioning holds promise for expanding the therapeutic armamentarium in AE, while remaining firmly hypothesis-generating at the current stage of evidence.

### 5.3 Integrated applications in prognostic prediction and rehabilitation management

The integration of multi-omics biomarkers with clinical and neuroimaging data may provide a future approach to prognostic prediction and rehabilitation planning in autoimmune encephalitis, but current evidence remains preliminary. Biomarkers derived from metabolomics and neuroimaging modalities, such as magnetic resonance imaging (MRI) and positron emission tomography (PET), provide insights into acute neuronal injury and recovery trajectories. For example, metabolic signatures associated with acute neuroinflammation and neuronal damage may help predict residual cognitive deficits, particularly in memory and executive functions, thereby informing the design of targeted cognitive rehabilitation programs (20). Advanced imaging biomarkers, including diffusion-weighted imaging (DWI) and apparent diffusion coefficient (ADC) values, correlate with disease severity and functional outcomes, serving as potential objective measures to tailor rehabilitation intensity (16). Moreover, longitudinal monitoring of cerebrospinal fluid (CSF) and serum biomarkers, such as neurofilament light chain (NfL) and glial fibrillary acidic protein (GFAP), may inform ongoing neuroaxonal injury and astrocytic activation, but their role in guiding therapeutic and rehabilitative adjustments requires prospective validation (21).

Importantly, multi-omics-derived molecular signatures may enable proof-of-concept predictive models for relapse risk by integrating baseline immune profiles and residual molecular abnormalities post-treatment. These models could eventually support personalized surveillance schedules and preemptive interventions, but their current utility is limited by small cohorts, retrospective designs, and insufficient external validation (15). The convergence of molecular prognostication with individualized rehabilitation therefore represents a research framework for AE rather than an established clinical pathway.

### 5.4 Future perspectives: computational integration and emerging technologies

The integration of advanced computational methods, including machine learning and network-based analyses, into AE multi-omics research represents an evolving frontier with both opportunities and limitations. Machine learning algorithms, including random forests, support vector machines, and deep neural networks, have been proposed to integrate heterogeneous multi-omics datasets and identify subtle molecular patterns that may predict treatment responses. In principle, AI-driven models could harmonize genomics, transcriptomics, proteomics, and clinical data to stratify patients into molecular subtypes with distinct therapeutic vulnerabilities. However, no such AI-integrated model has been developed or validated specifically for AE. The application of foundation models to AE remains purely theoretical and would require massive, harmonized multi-omics datasets that do not currently exist.

Similarly, computational drug repositioning approaches that compare AE-associated molecular signatures with drug-induced perturbation databases have nominated candidate compounds such as Janus kinase (JAK) inhibitors and sphingosine-1-phosphate (S1P) receptor modulators for AE subtypes characterized by specific immune dysregulation (23). These nominations are algorithmic predictions based on gene expression correlations; they require experimental validation in AE-specific cellular and animal models, followed by structured clinical trials, before any therapeutic relevance can be claimed. The integration of AI into AE multi-omics research must be approached with rigorous caution. Issues of algorithmic transparency, reproducibility, dataset bias, and the “black box” nature of deep learning models pose significant challenges. Emerging explainable AI (XAI) frameworks, such as SHAP (SHapley Additive exPlanations) and LIME (Local Interpretable Model-agnostic Explanations), offer promising avenues to enhance model transparency and facilitate clinical trust by quantifying individual feature contributions, though their application in AE multi-omics remains to be established. Any AI-derived biomarker or therapeutic prediction in AE should be regarded as a hypothesis-generating tool requiring extensive wet-lab and clinical validation, rather than an actionable clinical output.

## 6 Critical appraisal of current evidence and methodological limitations

While multi-omics integration holds substantial conceptual promise for advancing autoimmune encephalitis research, a balanced and critical assessment of the current evidence base is essential. The majority of multi-omics studies in AE are characterized by significant methodological limitations that temper enthusiasm and underscore the gap between research innovation and clinical implementation.

### 6.1 Sample size constraints and statistical power

Most published multi-omics AE studies involve fewer than 50 patients, with many comprising fewer than 30 participants. Such small cohort sizes severely limit statistical power, increase the risk of false-positive discoveries, and reduce the generalizability of findings to the broader AE population. For example, single-cell transcriptomic studies that identified clonal B-cell expansions and interferon pathway activation in anti-NMDAR encephalitis were conducted on 5–10 patients (14). While these studies generate important mechanistic hypotheses, the biomarker signatures and molecular networks identified require validation in substantially larger cohorts before they can be considered robust or clinically actionable. The “winner’s curse” phenomenon—wherein promising biomarkers from discovery cohorts fail to replicate in independent validation sets—is particularly pronounced in small-sample omics research.

### 6.2 Lack of replication and external validation

A critical weakness in the current literature is the near-absence of independent replication. Biomarker panels with reported AUC values exceeding 0.90 in discovery cohorts often fail to achieve comparable performance in independent validation sets. This phenomenon is particularly pronounced in small-sample omics research, where feature selection and model training on the same dataset inevitably lead to overfitting. To date, no multi-omics biomarker signature for AE diagnosis, prognosis, or treatment response has been prospectively validated in a multi-center, ethnically diverse cohort using pre-specified analytical protocols, underscoring a broader translational gap that has persisted despite the establishment of standardized clinical diagnostic frameworks (3).

### 6.3 Technical variability and batch effects

Multi-omics datasets are exquisitely sensitive to technical variation arising from different sequencing platforms, sample processing protocols, batch effects, and data analysis pipelines. CSF collection methods, processing delays, storage conditions, and batch-specific reagent lots can introduce substantial noise that may be mistaken for biological signal. Without rigorous standardization—such as the use of reference standards, quality control metrics, and harmonized protocols across centers—integration of multi-omics data from different studies remains problematic and may yield irreproducible conclusions. The lack of standardized multi-omics protocols specifically tailored for AE biospecimens (CSF, serum, PBMCs) further complicates cross-study comparisons.

### 6.4 Clinical translation barriers

Even when robust biomarkers are identified, multiple barriers impede clinical translation. These include: (i) high costs and lengthy turnaround times for multi-omics profiling, which are incompatible with the acute diagnostic time windows in AE; (ii) lack of regulatory-approved, clinically validated assays for most proposed biomarkers; (iii) insufficient evidence to guide how multi-omics data should alter clinical decision-making; and (iv) limited accessibility to advanced bioinformatics expertise in most clinical settings. Until these barriers are systematically addressed, multi-omics integration in AE will remain a research tool rather than a clinical reality.

### 6.5 Publication bias and the exploratory-confirmatory divide

The published literature likely overrepresents positive findings from small exploratory studies, while negative or inconclusive multi-omics results remain underreported. This publication bias distorts the perceived reliability of biomarker discoveries. Furthermore, many studies fail to adequately distinguish between exploratory hypothesis-generating analyses and confirmatory hypothesis-testing designs. The field would benefit from pre-registered study protocols, publicly available datasets, and mandatory independent validation phases before biomarkers are promoted as clinically relevant. The establishment of collaborative multi-center networks, such as the International Encephalitis Consortium, could facilitate large-scale prospective studies with standardized protocols and shared biospecimen repositories.

Overall, while multi-omics integration represents an important conceptual advance, the current AE evidence base is constrained by small studies, limited replication, technical heterogeneity, and uncertain clinical utility. Future research must prioritize large-scale collaborative cohorts, standardized protocols, pre-registered analyses, and rigorous prospective validation.

## 7 Conclusion

The integration of multi-omics approaches offers a promising but currently conceptual framework for understanding autoimmune encephalitis, a complex and heterogeneous neuroimmunological disorder. From an immunological perspective, this evolving paradigm—from reliance on single antibody detection toward comprehensive molecular characterization of adaptive and innate immune mechanisms—may eventually provide a systemic view that bridges critical gaps in diagnosis, prognosis, and therapeutic stratification. By synthesizing data across genomics, transcriptomics, proteomics, metabolomics, and immunomics, researchers have identified candidate biomarker signatures that may enhance diagnostic accuracy and elucidate the intricate molecular underpinnings driving clinical heterogeneity, including B-cell clonal expansion, demonstrated Tfh expansion with inferred Tfr imbalance, cytotoxic CD8+ T-cell-mediated neuronal injury in paraneoplastic/intracellular-antigen contexts, and complement-mediated tissue damage. At present, however, the most clinically informative integrations are those that combine candidate molecular biomarkers with conventional clinical, imaging, and electrophysiological data, whereas most single-cell, AI-driven, and treatment-stratification applications remain exploratory and hypothesis-generating. We have therefore deliberately avoided interpreting discovery-stage findings as clinically actionable conclusions.

Looking forward, realizing the potential of precision medicine in AE will require sustained collaborative efforts between clinicians, bioinformaticians, and immunologists, coupled with substantial investment in data infrastructure, standardization initiatives, and prospective validation studies. The establishment of international collaborative networks with shared biospecimen repositories and harmonized analytical pipelines will be essential to overcome the current limitations of small, single-center studies and accelerate the translation of multi-omics discoveries into clinical practice. Until such large-scale validation is achieved, multi-omics integration in AE should be regarded as a powerful hypothesis-generating and research-advancing tool rather than an actionable clinical framework.
